# Supplementary material for: Randomized feasibility trial of tele-yoga versus in-person yoga for treating chronic musculoskeletal pain in veterans
Source: BMC Complement Med Ther. 2026 Mar 17;26:153. doi: 10.1186/s12906-026-05345-y (PMC13107856; doi:10.1186/s12906-026-05345-y)
Supplement: Supplementary file 1 — Supplementary Material 1. [file 12906_2026_5345_MOESM1_ESM.docx]

**Additional File 1**

**Supplemental Figure 1: Diagram of Yoga Class Setup**


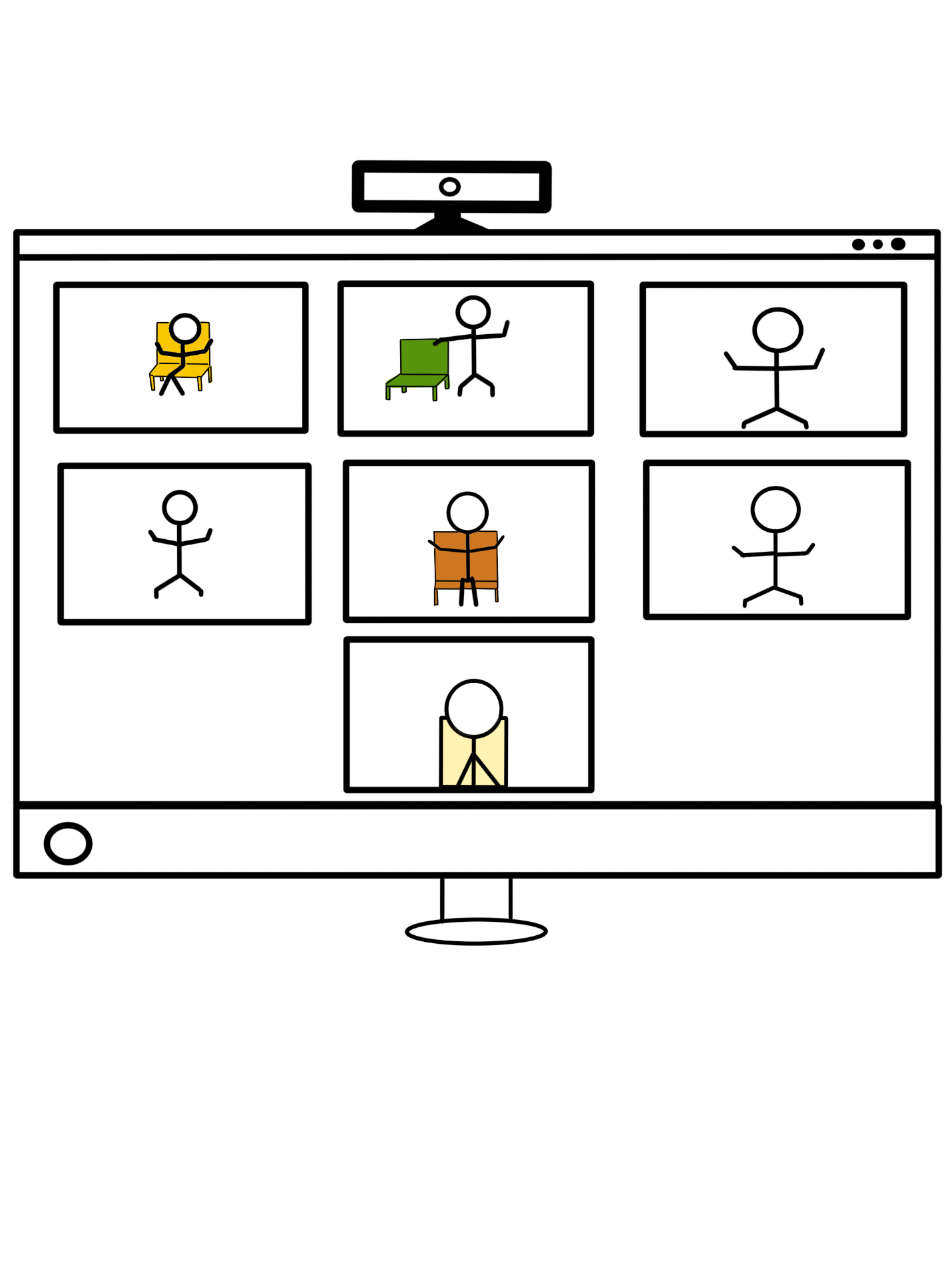


**2^nd^ Yoga Instructor, usually hidden from view**

**Veteran from Fremont, CA**

**Veteran from Santa Clara, CA**

**Veteran from Stockton, CA**

**Yoga Instructor in Palo Alto, CA**

**Veteran from San Jose, CA**

**Veteran from Salinas, CA**

**Supplemental Table 1: Phase 2 Screening and Baseline Scores**

|  | **In-person** | | | **Teleyoga** | | | **Group Difference** | |
| --- | --- | --- | --- | --- | --- | --- | --- | --- |
|  | N | Mean | SD | N | Mean | SD | Effect Size (Cohen's d) | p-val (2-tailed) |
| **SCREENING** |  |  |  |  |  |  |  |  |
| **SC_DVPRS_Q1_activity** | 16 | 6.063 | 1.569 | 18 | 5.889 | 1.530 | -0.112 | 0.747 |
| **SC_DVPRS_Q2_sleep** | 16 | 5.875 | 1.746 | 18 | 5.667 | 2.765 | -0.089 | 0.792 |
| **SC_DVPRS_Q3_mood** | 16 | 5.813 | 2.588 | 18 | 5.667 | 2.376 | -0.059 | 0.866 |
| **SC_DVPRS_Q4_stress** | 16 | 6.125 | 2.277 | 18 | 6.167 | 2.792 | 0.016 | 0.962 |
| **SC_PEG_Q1** | 16 | 6.000 | 2.280 | 18 | 5.889 | 2.139 | -0.050 | 0.885 |
| **SC_PEG_Q2** | 16 | 5.813 | 2.689 | 18 | 6.056 | 2.413 | 0.095 | 0.784 |
| **SC_PEG_Q3** | 16 | 6.000 | 2.394 | 18 | 5.889 | 1.997 | -0.051 | 0.885 |
| **SC_PEG_average** | 16 | 5.938 | 2.329 | 18 | 5.944 | 2.020 | 0.003 | 0.993 |
| **BASELINE** |  |  |  |  |  |  |  |  |
| **BL_PEG_average** | 16 | 5.688 | 1.926 | 18 | 6.241 | 1.763 | 0.300 | 0.391 |
| **BL_BPI_severity** | 16 | 4.953 | 1.980 | 18 | 5.458 | 1.563 | 0.285 | 0.420 |
| **BL_BPI_interference** | 16 | 4.854 | 2.309 | 18 | 5.142 | 2.141 | 0.130 | 0.710 |
| **BL_BDI2_total** | 16 | 6.000 | 6.501 | 18 | 5.333 | 5.269 | -0.113 | 0.747 |
| **BL_PROMIS6b_total** | 16 | 16.313 | 6.819 | 18 | 16.056 | 6.235 | -0.039 | 0.910 |

Supplemental Table 1 shows the screening and baseline characteristics of Teleyoga and In-Person groups using means and standard deviations for the continuous, and frequencies and percentages for categorical variables. Although unnecessary given our RCT design, group differences were examined using two-sample t-test for continuous and chi-square test for categorical variables. The two groups did not show significant differences in any of these baseline variables.

**Supplemental Table 2: Attrition rates**

| **Protocol Type** | **Attrition** | | |
| --- | --- | --- | --- |
|  | In-Person PP n=11 ITT n=16 | Teleyoga PP n=16 ITT n=18 | Combined PP n=27 ITT n=34 |
| PP | 3 (27%) | 3 (19%) | 6 (22%) |
| ITT | 8 (50%) | 3 (17%) | 11 (32%) |

PP – Per Protocol – Participants who received the intervention and completed EOT assessments
ITT – Intention to Treat – Participants who were randomized to the study regardless of whether they received any intervention or completed EOT assessments

**Supplemental Table 3: Teacher Fidelity to the Yoga Protocol**

| **Yoga Protocol Fidelity** | |
| --- | --- |
| IP Fidelity | 91% |
| TY Fidelity | 90% |
| Overall Fidelity | 91% |

**Supplemental Table 4: Missing Data (PEG and Promis-6b)**

|  | Teleyoga (n=18) | | In-person (n=16) | |
| --- | --- | --- | --- | --- |
|  | Baseline n items (%) | Post-Treatment n (%) | Baseline n (%) | Post-Treatment n (%) |
| Missing Data | 6 (4.2) | 7 (4.9) | 7 (8) | 14 (15.9) |

Baseline (BL) and EOT missing data for the PEG and Promis-6b combined.

**Supplemental Table 5: Symptom improvement based on average scores (Per Protocol)**

|  |  | Teleyoga  (n=13) | In Person  (n=8) | Difference  Teleyoga vs In-Person |
| --- | --- | --- | --- | --- |
| PEG average (SD) | Baseline  EOT  Change | 6.28 (1.87)  4.79 (1.53)  -1.49 | 5.50 (1.62)  4.54 (2.27)  -.96 | .53 |
| BPI severity (SD) | Baseline  EOT  Change | 5.62 (1.58)  4.15 (1.51)  -1.47 | 4.28 (1.90)  4.28 (2.0)  -.16 | 1.31 |
| BPI interference (SD) | Baseline  EOT  Change | 5.23 (2.19)  4.15 (2.21)  -1.08 | 3.93 (2.0)  4.29 (2.53)  +.36 | 1.44 |
| PROMIS6b total (SD) | Baseline  EOT  Change | 14.23 (5.49)  13.42 (5.77)  -.81 | 14.25 (7.50)  16.67 (5.62)  +2.42 | 3.23 |
| BDI total (SD) | Baseline  EOT  Change | 5 (4.17)  5 (4.67)  0 | 4.63 (5.1)  3.71 (3.57)  -.92 | -.92 |

**Experimental Measures**

**Supplemental Table 6: Homework (HW) Practice and HW Log**

|  | **In-Person (n=8)** | **Teleyoga (n=13)** |
| --- | --- | --- |
| Completed any weekly HW log: | 6 (75%) | 9 (69%) |
| Did ≥1 HW practice over 12 weeks | 5 (63%) | 9 (69%) |
| Range (in days/week) of practice | 0-6 | 0-6 |
| Range (in minutes) of practice | 0-2200 | 0-1694 |
| Average weeks with ≥ 1 practice | 1.6 | 5.31 (44%) p=4.5 |
| Average weekly minutes practiced | 49 | 39 p=45 |
| Total days practiced | 159 | 252 |
| Total minutes practiced | 6595 | 6483 |
| Used homework packet | 7 | 9 |
| Reported use of youtube videos | 2 | 2 |
| Breathing practice | 7 | 9 |
| Other homework practice | 1 | 2 |
| Unknown type of homework | 2 | 3 |

**Supplemental Figure 2: Usage Data for Homework Videos from Youtube**

**
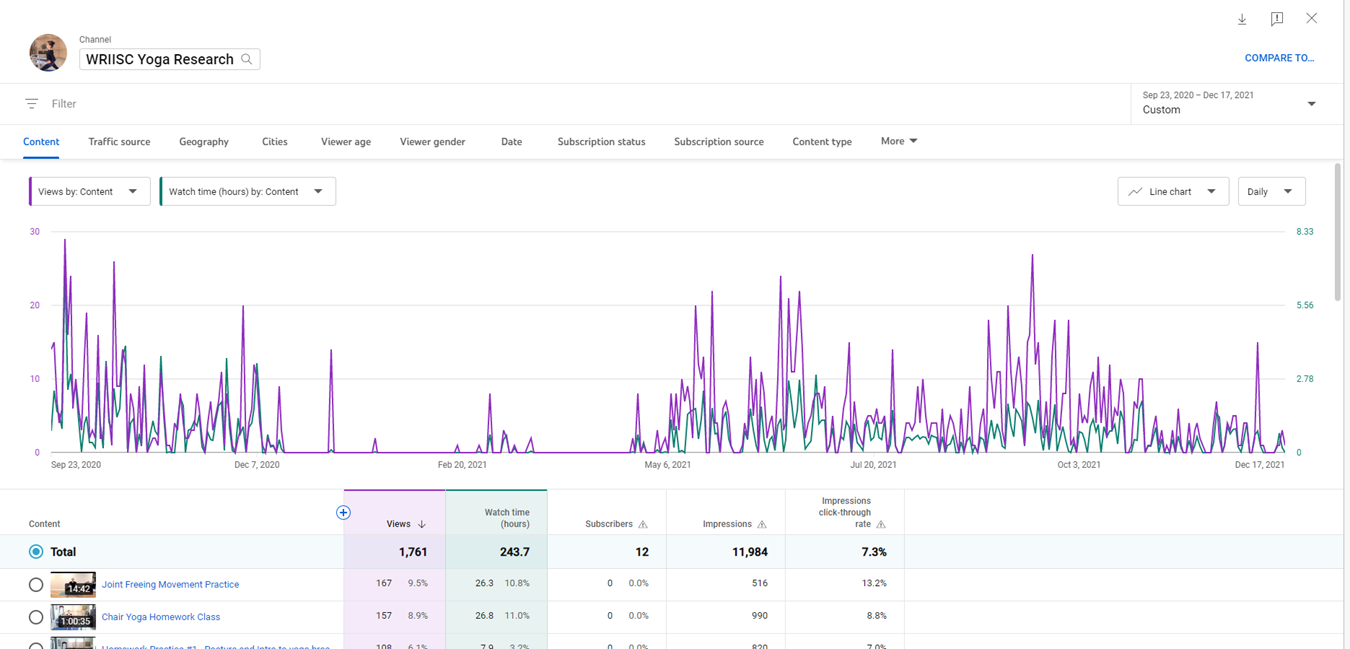
**

**Supplemental Table 7: Medication Usage at Baseline**

| **Reported Medication Usage at Baseline** | | | | | | |
| --- | --- | --- | --- | --- | --- | --- |
| Medication Type | Total (n=34) | | In-Person n=16 | | Teleyoga n=18 | |
|  | n | % | n | % | n | % |
| Anti-hypertensive | 10 | 29% | 2 | 13% | 8 | 44% |
| OTC Pain relief/NSAID | 9 | 26% | 4 | 25% | 5 | 28% |
| Statin | 8 | 24% | 0 | 0% | 8 | 44% |
| Anti-depressant | 5 | 15% | 2 | 13% | 3 | 17% |
| Gabapentin | 4 | 12% | 0 | 0% | 4 | 22% |
| Prostate Meds | 4 | 12% | 0 | 0% | 4 | 22% |
| No Medication Listed | 4 | 12% | 2 | 13% | 2 | 11% |
| Beta blocker | 2 | 6% | 0 | 0% | 2 | 11% |
| Thyroid Meds | 2 | 6% | 0 | 0% | 2 | 11% |
| Anti-Gout | 2 | 6% | 0 | 0% | 2 | 11% |
| Opioid | 1 | 3% | 1 | 6% | 0 | 0% |
| Skeletal muscle relaxant | 1 | 3% | 1 | 6% | 0 | 0% |

**Supplemental Table 8: Non-Yoga Therapies at Baseline**

| **Reported Therapy Usage at Baseline** | | | | | | |
| --- | --- | --- | --- | --- | --- | --- |
| Therapy Type | Total (n=34) | | In-Person (n=16) | | Teleyoga (n=18) | |
|  | n | % | n | % | n | % |
| Physical Therapy | 4 | 12% | 3 | 19% | 1 | 6% |
| Chiropractic | 4 | 12% | 3 | 19% | 1 | 6% |
| Acupuncture | 4 | 12% | 3 | 19% | 1 | 6% |
| Psychotherapy | 1 | 3% | 0 | 0% | 1 | 6% |
| Art Therapy | 1 | 3% | 1 | 6% | 0 | 0% |
| TNS Unit | 2 | 6% | 1 | 6% | 1 | 6% |
| H-Wave | 1 | 3% | 1 | 6% | 0 | 0% |
| Compression | 1 | 3% | 1 | 6% | 0 | 0% |
| Psychotherapy | 3 | 9% | 1 | 6% | 3 | 17% |
| Water/Pool therapy | 1 | 3% | 0 | 0% | 1 | 6% |
| Exercise (includes sports) | 6 | 18% | 4 | 25% | 2 | 11% |
| No Therapy Listed | 13 | 38% | 5 | 31% | 8 | 44% |
| Ice | 1 | 3% | 1 | 6% | 0 | 0% |
| Biofeedback | 1 | 3% | 1 | 6% | 0 | 0% |
| Podiatry | 1 | 3% | 1 | 6% | 0 | 0% |
| Whole Health Coaching | 1 | 3% | 1 | 6% | 0 | 0% |
| No Form Submitted | 3 | 9% | 2 | 13% | 1 | 6% |

**Supplemental Table 9: Yoga Protocol Summary**

| **Target** | **Yoga Tool** | **Weekly Class Themes** |
| --- | --- | --- |
| Breath and sensation awareness | Natural breath, body scan, thoracic diaphragm breathing | 1. Breath 2. Self-Compassion and self-love 3. Starting where you are 4. Start small and move slowly - with comfort. 5. Linking movement with breath 6. Focus on the positive – what is going right for us in this moment. 7. Notice how pain has become familiar - like a protective shell - but you don't need it, let it go. 8. Focus on slow breath - in and out - throughout the yoga practice. 9. Have fun, laugh at yourself and your teacher - love who you are right now. 10. Breath and movement as one 11. Yoga is the ability to direct attention to a chosen object without wavering - Yoga Sutra 1.2 - reemphasize living with compassion towards yourself and others. 12. Review how to bring what we’ve learned in class into daily living and options for yoga in the community |
| Balancing, calming, activating breath practices | Even (Sama Vritti), cooling (Sheetkari), alternate nostril (Nadi Shodhana), ocean (Ujayii), lion’s (Simha), humming (Brahmari), skull shining (Kapalabhati), Breath of Joy, Soft Belly breath |  |
| Posture and alignment | Seated or standing mountain (Tadasana) |  |
| Musculoskeletal strength, balance and flexibility, Posture, alignment, moving with the pace of the breath | Warrior (Virabhadrasana) I & II, 5 pointed start (Utthita Tadasana), chair (Utkatasana), horse (Vatayanasana), upward salute (Urdhva Hastasana) with lateral bend, wide leg forward fold (Prasarita Padottanasana), pyramid (Parvostanasana), tree (Vrikshasana), Heel rise in mountain balance, modified reclining bound angle (Supta Padangusthasana), figure 4 stretch (Ardha Matsyendrasana), side plank (Vasisthasana), modified locust (Salabhasana), child’s pose (Balasana), Bent Knee/Tabletop internal oblique strengthen, modified, bridge (Setu Bandha Sarvangasana) |  |
| Calming | Crocodile (Makarasana), bent knee spinal twist (Jathara Parivartanasana), legs up the wall (on a chair) (Viparita Karani), corpse (Savasana) |  |
| Hand Gestures | Prayer (Anjali), sun (Surya), deer (Mrigi) Mudras |  |
