# Supplementary material for: Randomized feasibility trial of tele-yoga versus in-person yoga for treating chronic musculoskeletal pain in veterans
Source: BMC Complement Med Ther. 2026 Mar 17;26:153. doi: 10.1186/s12906-026-05345-y (PMC13107856; doi:10.1186/s12906-026-05345-y)
Supplement: Supplementary file 2 — Supplementary Material 2. [file 12906_2026_5345_MOESM2_ESM.docx]

**Additional File 2**

**Yoga Teacher Hiring and Training**

All yoga teacher applicants were required to be Yoga Alliance registered yoga teachers with a minimum of a 200-hour yoga teacher certification and at least some teaching experience.

During the hiring process the primary yoga instructor interviewed each eligible applicant by phone and then held teaching auditions for the top candidates using Zoom. Each finalist was provided with a 30-minute sample class protocol and was asked to let us know when they were ready to teach. We assessed each finalist using a pre-determined list of criteria:

- Fidelity to the protocol
- Tone of voice
- Clarity of instruction
- Attention to safety during instruction
- Adequate teaching space (room and lighting)
- Availability (flexible to meet participant scheduling needs)
- Ability to drive to Palo Alto for in-person classes

Each item received a score on a scale of 1-5 and the top scoring instructors were offered the job.

Once hired, the yoga instructors observed the primary yoga instructor deliver the protocol during Phase 1, Cohort 2. The 12-week protocol was reviewed and further revised by the yoga instructor team during weekly meetings. During this process we simplified the supine and pose postures for ease of instruction and removed some of the variations on the poses to minimize the need for participants to look at the screen for instruction.

Each hired yoga instructor led the other instructors in the yoga sequences prior to commencing treatment in Phase 2 to assure fidelity to the protocol.
